# Bmi1 inhibition enhances the sensitivity of pancreatic cancer cells to gemcitabine

**DOI:** 10.18632/oncotarget.9293

**Published:** 2016-05-11

**Authors:** Tao Yin, Zhengle Zhang, Bin Cao, Qingke Duan, Pengfei Shi, Hengqiang Zhao, Soriba Naby Camara, Qiang Shen, Chunyou Wang

**Affiliations:** ^1^ Department of Pancreatic Surgery, Union Hospital, Tongji Medical College, Huazhong University of Science and Technology, Wuhan, P.R. China; ^2^ Department of Hepatobiliary Surgery, Union Hospital, Tongji Medical College, Huazhong University of Science and Technology, Wuhan, P.R. China; ^3^ Department of Clinical Cancer Prevention, The University of Texas MD Anderson Cancer Center, Houston, TX, USA

**Keywords:** pancreatic cancer, Bmi1, gemcitabine, chemotherapy, efficacy

## Abstract

As the standard therapy for pancreatic cancer, gemcitabine shows limited efficacy in pancreatic cancer patients because of chemoresistance. Aberrant expression of Bmi1 has been reported to activate multiple growth-regulatory pathways and confer anti-apoptotic abilities to many cancer cells. However, the role of Bmi1 in response of pancreatic cancer cells towards gemcitabine resistance remains elusive. In this study, we found that certain dose of gemcitabine treatment induced Bmi1 expression in pancreatic cancer cells. Knockdown of Bmi1 enhanced ROS production and promoted the cytotoxic effect of gemcitabine. The increased oxidative stress upon gemcitabine treatment could disrupt mitochondrial membrane and decrease mitochondrial membrane potential, eventually leading to apoptosis. Bmi1 inhibition also suppressed the activation of NF-κB signaling and the expressions of downstream molecules in pancreatic cancer cells treated with gemcitabine. Moreover, we observed Bmi1 inhibition sensitized the pancreatic xenograft tumors to gemcitabine in vivo. Taken together, our study demonstrated that Bmi1 could decrease the sensitivity of pancreatic cancer cells to gemcitabine through increasing oxidative stress and inhibiting NF-κB signaling, thus Bmi1 may serve as a promising target for sensitizing pancreatic cancer cells to chemotherapy.

## INTRODUCTION

Pancreatic cancer is a highly malignant digestive system tumor. The incidence of pancreatic cancer has increased significantly in recent years. Unfortunately, the prognosis of pancreatic cancer is still very poor with a 5-year survival rate less than 5% [1, 2]. To date, there is no effective strategy for early detection of pancreatic cancer. Most pancreatic cancer patients are usually presented at an advanced stage once being diagnosed [3]. Currently, there are very few therapeutic options available for pancreatic cancer patients due to limited response or resistance to available systemic treatments [4].

At present, gemcitabine is the first-line chemotherapeutic agent for advanced and metastatic pancreatic cancers. However, low response rate to gemcitabine is common in clinic and the efficacy of gemcitabine is just less than 20% of treated patients [5, 6]. For patients underwent radical surgery and standard postoperative chemotherapy, the mean survival time is still short due to quick recurrence and metastasis. Chemotherapy does not show much benefit in preventing recurrence and prolonging the survival of pancreatic cancer patients [7]. A thorough understanding of the underlying mechanisms of pancreatic cancer chemoresistance will have imminent impact on developing strategies for sensitizing pancreatic cancer cells towards chemotherapy. Gemcitabine (2′, 2′-difluoro-2′-deoxycytidine; dFdC) is a deoxycytidine analog, whose cytotoxicity is depended on inducing apoptosis. After being uptaken into the cell, it is metabolized into the active form (dFdCTP) which can interfere with DNA synthesis and induce pro-apoptotic pathways [8]. However, aberrant activation of pro-survival pathways or molecules could decrease gemcitabine toxicity and cause drug resistance. These abnormal pathways or molecules may be potential targets for sensitizing pancreatic cancer to gemcitabine [9].

Bmi1, a component of the Polycomb Repressive Complex 1 (PRC1) which triggers histone H2A ubiquitylation and gene silencing [10], has been proved to be overexpressed and participated in the tumorigenesis of a variety of cancers including breast, lung, and leukemia, etc. [11–13]. Bmi1 functions as an oncogene and promotes the survival of cancer cells via regulating multiple growth-regulatory pathways [14]. Moreover, Bmi1 has been reported to confer anti-apoptotic ability and chemoresistance to cancer cells. [15, 16]. The abnormal expression of Bmi1 has been also reported to link disease progression and poor prognosis in pancreatic cancer patients [17]. However, the role of Bmi1 in response of pancreatic cancer cells towards chemotherapy and subsequent chemoresistance remains elusive. We hypothesized that aberrant expression of Bmi1 might closely correlate to the sensitivity of gemcitabine treatment in pancreatic cancer cells.

It has been established that chemotherapy produces reactive oxygen species (ROS) and induces oxidative stress in cancer cells. The level of oxidative stress may directly related to the chemotherapeutic efficacy, since excessive ROS will cause damage to intracellular structures, thus playing roles as modulators in initiation and execution of apoptosis [18]. A role of ROS in gemcitabine-induced cell growth inhibition has been reported previously in pancreatic cancer [19]. Whereas, Bmi1 could regulate oxidative stress by eradicating excessive ROS [20]. On the other hand, constitutive or acquired NF-κB activation is one of the known mechanisms of pancreatic cancer resistance against gemcitabine [6]. Modulation of NF-κB activity by pharmacological or genetic approaches may have therapeutic potentials [21, 22]. Meanwhile, Bmi1 has also been reported to activate the NF-κB signaling pathway and participate in the cancer progression [14]. Thus, we hypothesized that Bmi1 may suppress the sensitivity of pancreatic cancer cells towards gemcitabine treatment via regulating oxidative stress and NF-κB activity.

In this study, we explored a potential role for Bmi1 in mediating response that protects pancreatic cancer cells from the cytotoxic effect of gemcitabine via employing a loss of function approach through Bmi1 knockdown. Our results showed that Bmi1 inhibition sensitized pancreatic cancer to gemcitabine through aggravating oxidative stress and inhibiting NF-κB signaling.

## RESULTS

### Gemcitabine treatment induces Bmi1 expression in pancreatic cancer cells

**Figure 1:**
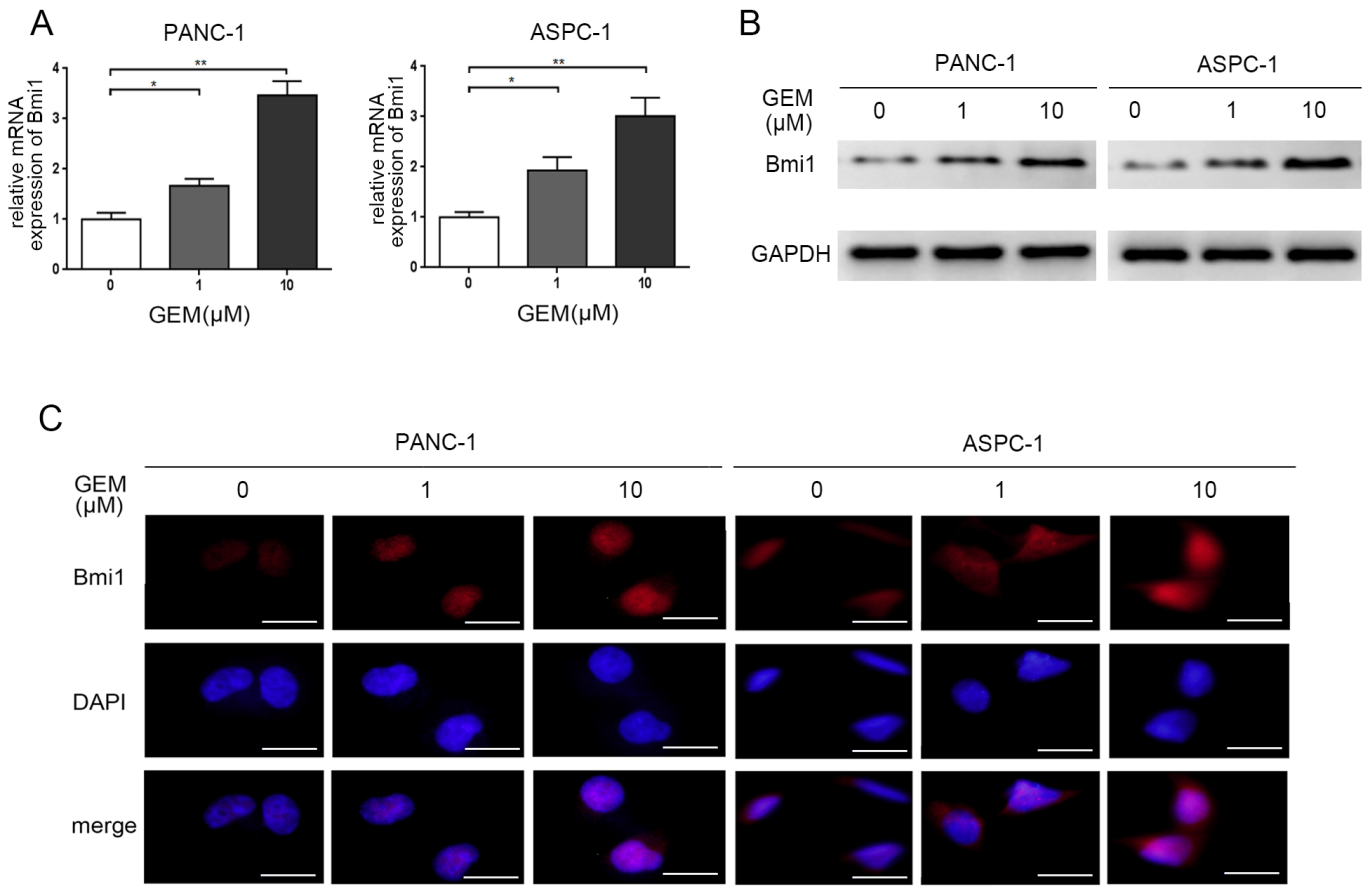
Gemcitabine treatment increases the expression of Bmi1 **A.** The expression levels of Bmi1 were detected by qRT-PCR in both two pancreatic cancer cells after different dose of gemcitabine treatments for 12h. **B.** The protein levels of Bmi1 were tested by Western Blot after different dose of gemcitabine treatments for 12h. **C.** Gemcitabine enhanced nuclear accumulation of Bmi1 by immunofluorescence staining. The graphs shown are representative results of three independently repeated experiments. Scale bar, 50 μm. *, P < 0.05; **, P < 0.01.

The endogenous expression of Bmi1 in pancreatic cancer has been reported to promote invasive and metastatic abilities in previous study [17]. To explore the potential role for Bmi1 towards chemotherapy, we detected the changes of Bmi1 expression in pancreatic cancer cells treated by gemcitabine. In our study, the PANC-1 and ASPC-1 pancreatic cancer cell lines were treated with different concentrations of gemcitabine, the alterations of Bmi1 expression were determined by quantitative real-time PCR, Western blot and immunofluorescence. We found an increased expression of Bmi1 at mRNA and protein levels after gemcitabine treatment at 1 μM and 10 μM concentrations for 12 h (Figure 1A–1B). Immunofluorescence further demonstrated an enhanced nuclear accumulation of Bmi1 after gemcitabine treatment (Figure 1C).

### Bmi1 facilitates the chemoresistance of pancreatic cancer cells to gemcitabine

**Figure 2:**
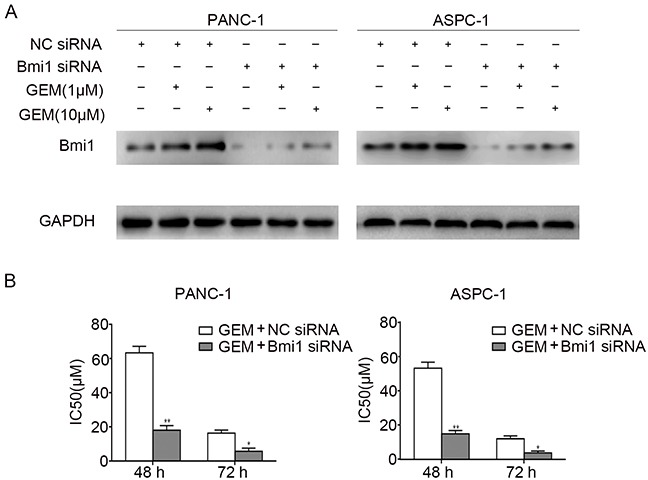
Bmi1 facilitates the chemoresistance of pancreatic cancer cells to gemcitabine **A.** The expressions of Bmi1 were examined by Western Blot in two pancreatic cancer cells transfected with Bmi1 siRNA and NC siRNA for 36 h respectively, followed by different dose of gemcitabine treatments for 12h. Graphs shown are representative result of three independent assays. **B.** After treatment as indicated, the IC50s of two cells to gemcitabine at 48 h and 72 h were valued by MTT assays. The data were showed from 3 parallel experiments *, P < 0.05; **, P < 0.01.

To examine whether Bmi1 induced by gemcitabine contribute to the drug resistance, we detected the IC50 values of gemcitabine for different time point by MTT assay. Loss-of-function of Bmi1 in pancreatic cancer cells was achieved by siRNA knockdown. As shown in Figure 2A, Bmi1 knockdown remarkably reduced Bmi1 protein level in both pancreatic cancer cells treated with gemcitabine. We then compared the sensitivity of pancreatic cancer cells to gemcitabine in combination with Bmi1 inhibition. As shown in Figure 2B, The IC50 values of gemcitabine at 48 h and 72 h were all significantly decreased after Bmi1 siRNA transfection followed by gemcitabine treatment. To further elucidate the role of Bmi1 in gemcitabine sensitivity, the Bmi1 expression vector pcDNA3.1-Bmi1 was transfected into pancreatic cancer cells. As shown in the result (Supplementary Figure S1), ectopic expression of Bmi1 dramatically increased the IC50s of gemcitabine at 48 h and 72 h in two pancreatic cancer cells. Taken together, our results suggested that Bmi1 promoted the chemoresistance of pancreatic cancer cells to gemcitabine.

### Gemcitabine and Bmi1 knockdown enhances ROS production and apoptosis of pancreatic cancer cells

**Figure 3:**
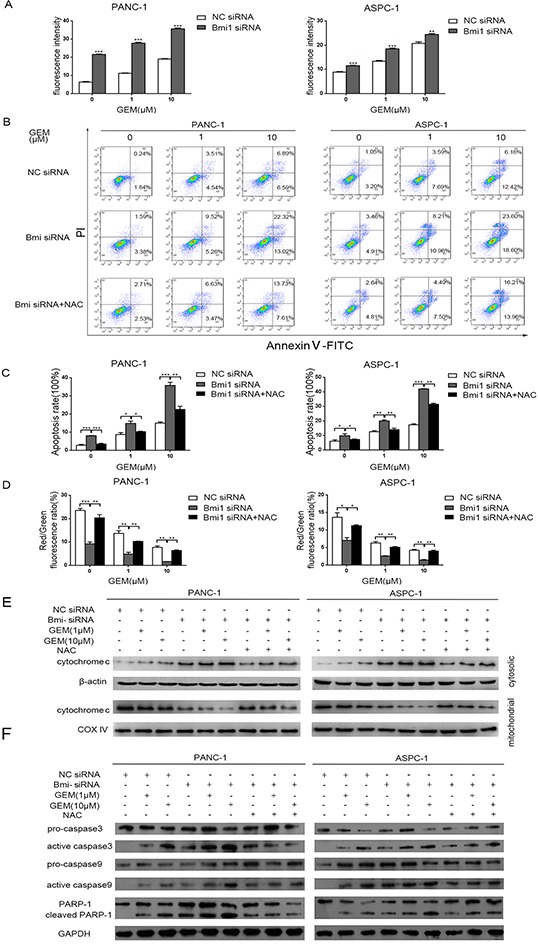
Downregulation of Bmi1 increases apoptosis of pancreatic cells mediated by ROS **A.** Pancreatic cancer intracellular ROS levels after Bmi1 inhibition combined with different dose of gemcitabine treatments were measured by DCFH-DA probe through fluorescence Spectrophotometer. **B.** Representative dot-plots illustrating the apoptotic status of PANC-1 and ASPC-1 cells using Annexin V-FITC/ PI method. The dot-plots in the right upper quadrant and the right lower quadrant were quantified as the percentage of apoptotic cells. **C.** The percentage of apoptosis of pancreatic cancer cells detected using Annexin V-FITC/ PI method as indicated above. **D.** The changes of mitochondrial membrane potential treated by Bmi1 knockdown, or additional NAC, combined with different dose of gemcitabine were valued by JC-1 method. **E.** The translocation of cytochrome C in pancreatic cancer cells treated with the indicated concentration of gemcitabine, Bmi1siRNA or additional NAC. The cytosolic and mitochondrial proteins were extracted and analyzed by western blotting. **F.** The expressions of proapoptotic proteins were detected by Western Blot after Bmi1 knockdown and different dose of gemcitabine treatment. Results shown are representative of three independent assays. Data in the graphs represent means ± SEM from triplicate repeated experiments. *, P < 0.05; **, P < 0.01, ***, P < 0.001.

Reactive Oxygen Species (ROS) induction is one of the mechanisms for gemcitabine to eradicate cancer cells [23]. Bmi1 was reported to regulate the redox balance and protect cell from the damage of oxidative stress [20]. To validate the role of ROS in the sensitization of gemcitabine after Bmi1 inhibition, we used DCFH-DA probe to detect ROS in pancreatic cancer cells. As a result, we detected increased ROS in pancreatic cancer cells treated with gemcitabine, and Bmi1 inhibition by siRNA transfection further enhanced ROS production. Our data suggested that Bmi1 inhibition synergized with gemcitabine treatment, and Bmi1 might play a suppressive role in gemcitabine-induced ROS production (Figure 3A).

Induction of apoptosis is a key mechanism for gemcitabine's antitumor effect. We observed that gemcitabine induced apoptosis of pancreatic cancer cells, corresponding with the accumulation of ROS. Moreover, this apoptosis and ROS production in pancreatic cancer cells was further increased with Bmi1 knockdown. Whereas, ROS scavenger NAC (N-acetyl-L-cysteine) could rescued the apoptosis induced by gemcitabine combined with Bmi1 knockdown (Figure 3B–3C).

Mitochondria are important inducer of apoptosis and mitochondrial membrane potential (Δψm) is an important index for evaluating apoptosis. Damage of mitochondrial membrane may lead to increased mitochondrial membrane permeability, reduced Δψm and the release of pro-apoptotic factors into the cytosol. To further explore the mechanisms of increased apoptosis of pancreatic cancer cells, we determined the Δψm of pancreatic cancer cells using a JC-1 method after gemcitabine treatment and Bmi1 inhibition. As a result, we found significant decrease of Δψm in cells treated with gemcitabine and Bmi1 siRNA. Correspondingly, NAC treatment increased Δψm by scavenging ROS (Figure 3D). These results suggested that Bmi1 inhibition promoted excessive ROS production induced by gemcitabine treatment, leading to mitochondrial membrane damage and triggering of apoptosis.

To further validate the expression of apoptotic proteins, we found cytochrome C increased in the cytosol and decreased in the mitochondria after treated with gemcitabine and Bmi1 siRNA. In addition, NAC inhibited the release of cytochrome C from mitochondria into the cytosol (Figure 3E). Accordingly, gemcitabine and Bmi1 siRNA enhanced active caspase-3, active caspase-9 and cleaved poly (ADP-ribose) polymerase-1 in pancreatic cancer cells, and these enhancements could be also suppressed by NAC treatment (Figure 3F). Altogether, our data suggested that Bmi1 inhibition might promote the release of cytochrome c into the cytosol in gemcitabine-treated pancreatic cancer cells, leading to apoptotic cascade at least in part by enhancing ROS production.

### Bmi1 knockdown inhibits the expression of antioxidant genes

**Figure 4:**
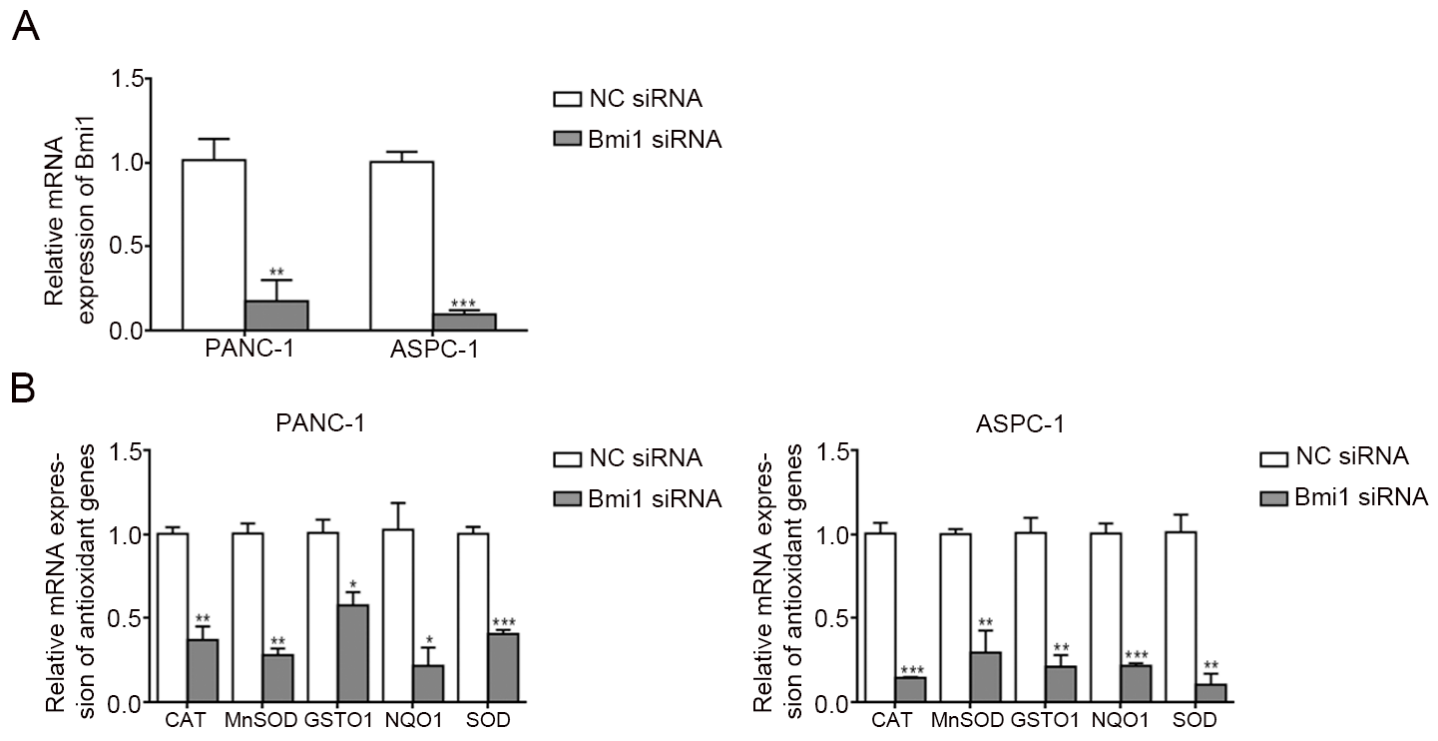
Bmi1 knockdown alters the expression of antioxidant genes **A.** QRT-PCR revealed the mRNA level of Bmi1 significantly decreased transfected with Bmi1 siRNA. **B.** The mRNA levels of CAT, MnSOD, GSTO1, NQO1 and SOD after Bmi1 knockdown were tested by qRT-PCR. *, P < 0.05; **, P < 0.01, ***, P < 0.001.

Bmi1 has been reported to play an antioxidant role by regulating antioxidant defenses [20]. To explore the underlying mechanisms of enhanced ROS production after Bmi1 knockdown in pancreatic cancer cells, we compared the expression of antioxidant genes before and after Bmi1 siRNA transfection through quantitative real-time PCR. We found Bmi1 siRNA inhibited Bmi1 expression in both two pancreatic cancer cells effectively (Figure 4A). After Bmi1 knockdown, the expression of antioxidant genes including CAT, MnSOD, GSTO1, NQO1 and SOD decreased significantly in pancreatic cancer cells (Figure 4B).

### Bmi1 knockdown inhibits the activity of NF-κB in pancreatic cancer cells treated with gemcitabine

**Figure 5:**
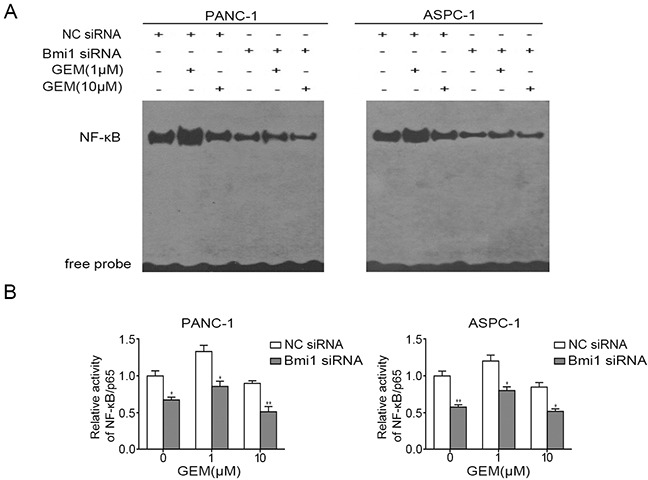
Bmi1 knockdown inhibits the activity of NF-κB treated with gemcitabine **A.** Pancreatic cancer cells were transfected with siRNA and treated with gemcitabine, the DNA binding activities of NF-κB in pancreatic cancer cells were detected by EMSA. **B.** After treatment as indicated, the activities of p65 subunit of NF-κB entering into the nucleus were further examined by NF-κB ELISA assay. Results shown are representative of three independent assays. *, P < 0.05; **, P < 0.01.

Activation of NF-κB is one of the mechanisms for pancreatic cancer to resist chemotherapy [6]. To further determine whether the enhanced sensitivity to gemcitabine after Bmi1 inhibition is correlated with the changes of NF-κB activity in pancreatic cancer cells, we compared the status of NF-κB in pancreatic cancer cells through EMSA. In our result, low concentration of gemcitabine could induce activation of NF-κB in both cell lines (Figure 5A), while this activation was significantly inhibited after Bmi1 siRNA transfection. The activation of NF-κB was further verified by NF-κB ELISA assay, which detected the activated p65 subunit of NF-κB entering into the nucleus. The results also showed that Bmi1 inhibition suppressed the activation of NF-κB treated with gemcitabine (Figure 5B).

We further detected the effect of Bmi1 inhibition on the NF-κB signaling cascade. Western blot analysis showed Bmi1 knockdown obviously inhibited the expression of phosphorylated IKKα/β and phosphorylated IκBα which played important roles in NF-κB activation. Furthermore, we also observed a decrease in NF-κB/p65 levels both in nuclear and cytoplasmic fractions of pancreatic cancer cells after Bmi1 inhibition by immunoblot analysis of subcellular fractionation (Supplementary Figure S2). Altogether, our results suggested Bmi1 knockdown inhibited the activation of NF-κB signaling cascade in pancreatic cancer cells treated with gemcitabine.

### Bmi1 inhibition sensitizes the pancreatic cancer cell to gemcitabine via down-regulation of NF-κB target genes

**Figure 6:**
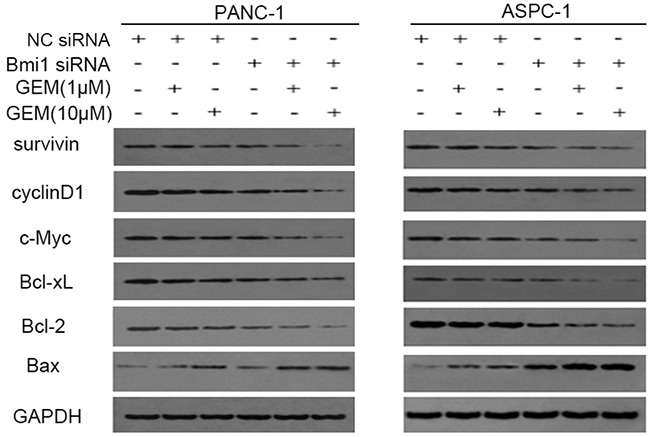
Bmi1 inhibition regulates NF-κB downstream genes Pancreatic cancer cells transfected with Bmi1 siRNA were treated with gemcitabine. The alterations of NF-κB downstream genes including survivin, Bcl-xL, Bcl-2 and Bax which were related with apoptosis; cyclinD1 and c-Myc which were related with proliferation were tested by Western Blot after Bmi1 knockdown, combined with different dose of gemcitabine treatment. Graphs shown are representative results of three repeated assays.

NF-κB is involved in chemoresistance and apoptosis via regulating a series of survival-related genes. We sought to determine the changed genes downstream of NF-κB, which were involved in regulating apoptosis or proliferation of pancreatic cancer cells. We found that combination treatment with gemcitabine and Bmi1siRNA suppressed the expression of survivin, Bcl-xL and Bcl-2, which were involved in inhibiting apoptosis. On the contrary, the expression of Bax, a gene promoting apoptosis, increased significantly after co-treatment with gemcitabine and Bmi1 siRNA. We also found that cyclinD1 and c-Myc, which participated in regulating proliferation, were also suppressed after Bmi1 inhibition (Figure 6).

### Bmi1 inhibition sensitizes the pancreatic xenograft tumors to gemcitabine

The effect of gemcitabine treatment in combination with Bmi1 inhibition on the growth of pancreatic xenograft tumors was further determined *in vivo*. Pancreatic cancer xenograft model was established by subcutaneous injection of pancreatic cancer cells into the right flank of nude mice. The treatments were initiated after comparable tumor volumes reached in tumor-bearing mice (approximately 120 mm^3^) as described in materials and methods.

**Figure 7:**
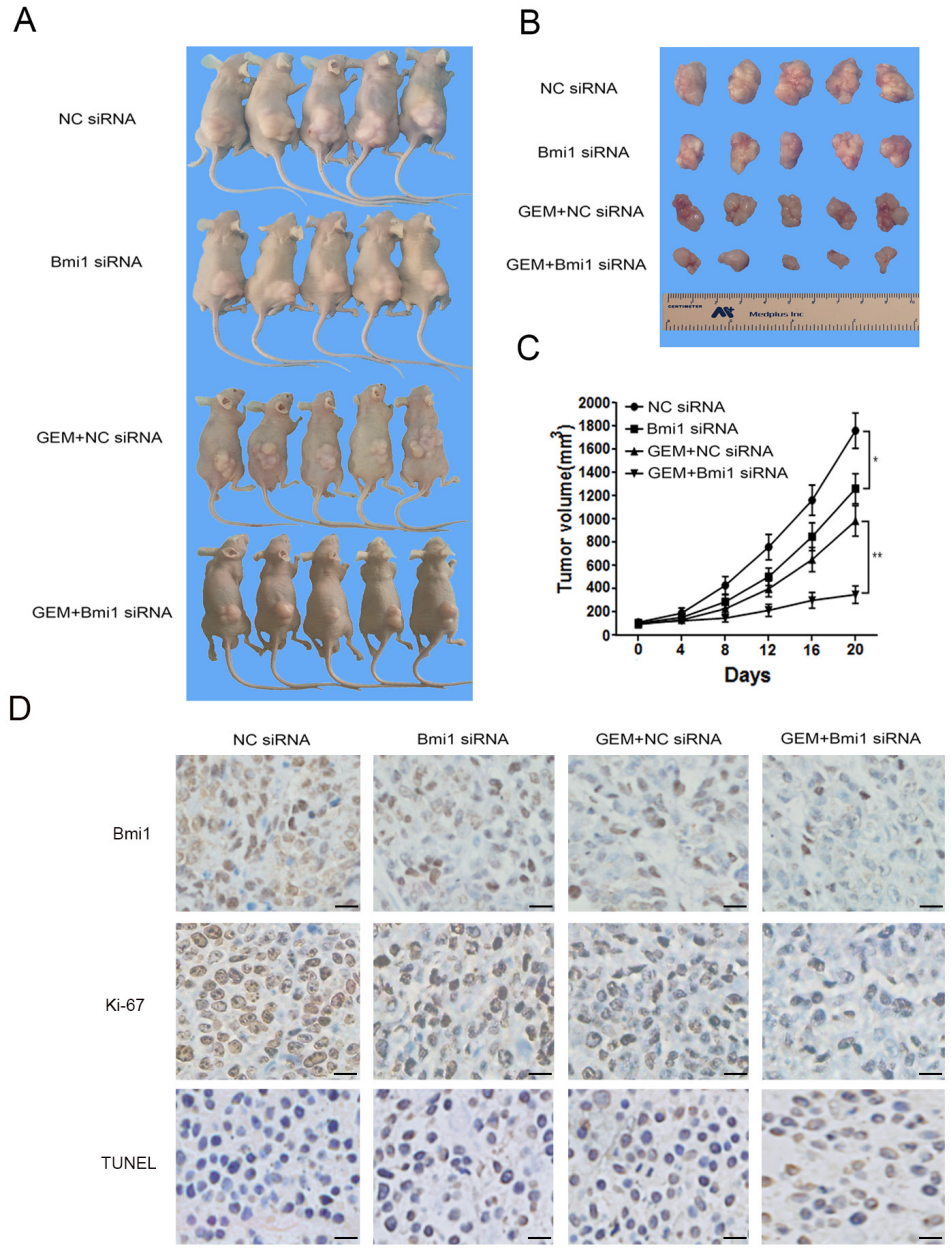
Bmi1 inhibition sensitizes the effect of gemcitabine in pancreatic xenograft tumors **A–B.** PANC-1 cells were subcutaneously injected into the right flank of nude mice. After comparable tumor volumes were reached approximately 120 mm^3^, the mice were randomly divided into Bmi1 siRNA, NC siRNA, GEM+Bmi1 siRNA and GEM+NC siRNA groups, respectively, and treated as described in section 4. Tumor size was measured after about 20 days' treatment. **C.** Tumor volumes were measured every 4 days. Tumor growth curve was drawn according the tumor volume measured. **D.** The representative tumor tissue sections from xenografts in different treatment group were analyzed by immunohistochemistry for the expression of Bmi1 and the proliferation marker Ki-67. The apoptotic cells were stained by TUNEL agent. Scale bar, 20 μm. *P<0.05; **, P < 0.01.

As showed in Figure 7A–7C, compared with NC siRNA group, the growth rate and size of tumors in Bmi1 siRNA group were significantly reduced. When combined with gemcitabine chemotherapy, in vivo silencing of Bmi1 also dramatically decreased the tumor's growth compared with controls. To further determine the efficacy of the Bmi1 siRNA transfection in vivo, immunohistochemistry was performed to analyze the Bmi1 expression in tumor tissue. We found decreased expression of Bmi1 after Bmi1 siRNA transfection both in gemcitabine treated and untreated groups (Figure 7D). Moreover, immunohistochemistrical staining of proliferation and apoptotic markers showed decreased Ki-67 index and increased TUNEL staining in group treated with gemcitabine in combination with Bmi1 siRNA (Figure 7D).

## DISCUSSION

As an oncogene, Bmi1 is abundantly expressed in many kinds of cancers and is correlated with multiple behaviors including tumorigenicity, drug resistance and cancer recurrence [24–26]. In our study, we found that a certain concentration range of gemcitabine promoted Bmi1 expression in pancreatic cancer cells. Moreover, the chemotherapeutic sensitivity of pancreatic cancer to gemcitabine can be increased by Bmi1 inhibition both in vitro and in vivo. Bmi1 may participate in regulating the chemosensitivity of pancreatic cancer to chemotherapy. Nevertheless, we also found that high dose (e.g. 50μM) of gemcitabine could inhibit Bmi1 expression (data not shown), which might account for the reduced Bmi1 staining of tumor tissues in nude mice received high gemcitabine chemotherapy. Gemcitabine has been proved to exert different effects on pancreatic cancer cells in a dose and time dependent manner by inducing different signaling pathways [21, 27], whereas Bmi1 expression can also be affected by such specific signaling molecules [28]. Thus, it is tempting to speculate that different signaling pathways induced by low or high concentrations of gemcitabine treatment may exert promoting or inhibiting role on Bmi1 expression in pancreatic cancer cells.

Chemotherapeutic drug can induce ROS and produce oxidative stress, which may play roles in determining the efficacy of chemotherapy [29]. It was reported that ROS induced by gemcitabine served as one of the mechanisms for inhibiting pancreatic cancer growth [19, 29]. In our study, Bmi1 inhibition aggravated the production of ROS in pancreatic cancer cells induced by gemcitabine treatment. Correspondingly, we also verified the increased apoptosis of pancreatic cancer cells treated with gemcitabine combined with Bmi1 inhibition, and this enhanced apoptosis could be inhibited by ROS scavenger. The results indicated that Bmi1 may protect pancreatic cancer cells from apoptosis by removing excessive ROS induced by gemcitabine treatment. Mitochondria are important inducer of apoptosis and ROS target [30]. Mitochondrial Δψm is not only a valuable index of apoptosis, but also an indicator of mitochondrial permeability [31]. Our results showed Bmi1 inhibition decreased Δψm induced by gemcitabine, the effect of which could also be suppressed by NAC. Besides, we also found enhanced translocation of cytochrome C from mitochondria to the cytosol of pancreatic cancer cells treated with gemcitabine and Bmi1 siRNA. Accordingly, the apoptotic cascade downstream of cytochrome C including activated caspase 3, activated caspase 9 and cleaved PARP-1 were also increased. Taken together, our results suggested that Bmi1 inhibition further enhanced the gemcitabine-induced ROS and caused damage of mitochondria membrane, leading to an increase in permeability of mitochondrial membrane and apoptosis.

Elevated levels of oxidative stress have been proved in almost all cancers and affect many aspects of malignant behavior, including tumor initiation and progression. Cancer cells thus develop multiple ways for adapting ROS for their survival [32]. Bmi1 has been reported to maintain function of mitochondria which are main producer of ROS [33]. In our study, we found that Bmi1 might protect pancreatic cancer cells from oxidative stress through regulating a series of antioxidant enzymes. Thus, to elucidate and destroy the mechanisms underlying ROS metabolism may have potential significance for increasing the sensitivity of pancreatic cancer cells to chemotherapy.

Aberrant activation of NF-κB has been reported as an important mechanism of drug resistance [34]. NF-κB, exhibiting a constitutively active state in pancreatic cancer, is proved to play an anti-apoptotic role and promote aberrant tumor growth [35]. In our study, we revealed that low dose of gemcitabine treatment could stimulate the activation of NF-κB, and the inductive effect could be weakened when combined with Bmi1 knockdown. Furthermore, Bmi1 inhibition suppressed the activation of upstream molecules including IKKα/β and IκBα, which was coincided with the previous report [14]. Furthermore, the NF-κB downstream molecules, which involved in apoptosis repression and growth regulation, were also repressed when treated with gemcitabine and Bmi1 siRNA. These results suggested that Bmi1 might participate in the activation of NF-κB, and Bmi1 inhibition blocked the activation of NF-κB signaling cascade induced by gemcitabine treatment. Nevertheless, we also observed that high dose of gemcitabine suppressed NF-κB activation. One of the possible mechanisms accounting for this phenomenon may be the excessive accumulation of ROS induced by high dose gemcitabine treatment, since ROS has been showed to exert two-side effects on NF-κB signaling. A limited dose of ROS could activate NF-κB signaling, whereas it has negative effects on the signaling above a certain threshold [36].

**Figure 8:**
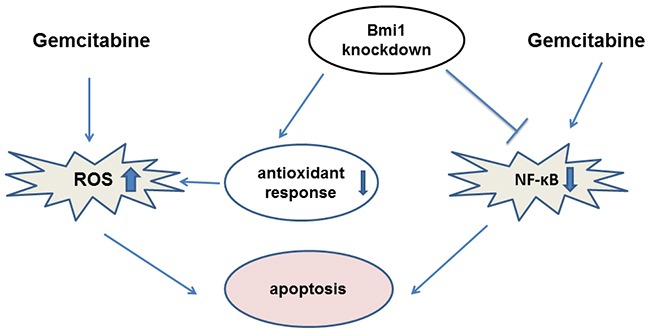
A schematic model illuminating the potential roles of Bmi1 knockdown in enhancing the sensitivity of pancreatic cancer cells to gemcitabine

In summary, our study demonstrated that gemcitabine treatment combined with Bmi1 silencing could improve gemcitabine chemosensitivity by increasing intracellular ROS and inhibiting of NF-κB activity (Figure 8). Our data will provide a potentially new strategy for improvement of the chemosensitivity of gemcitabine in treating pancreatic cancer.

## MATERIALS AND METHODS

### Cell culture

The pancreatic cancer cell lines PNAC-1 and ASPC-1 were originated from ATCC, and were cultured in Dulbecco Modified Eagle Medium supplemented with 10% fetal bovine serum, penicillin (100U/ml) and streptomycin (100μg/mL) at 37°C with 5% CO2.

### SiRNA construction and cell transfection

The Bmi1 siRNA was designed and synthesized by GenePharma Co., Ltd., Shanghai, China. The double-strand Bmi1 siRNA (sense 5′-AUGAAGAGAAGAAGGGAUUTT-3′, antisense 5′-AAUCCCUUCUUCUCUUCAUTT-3′) and negative control siRNA (NC siRNA: sense 5′-UUCUCCGAACGUGUCACGUTT-3′; antisense 5′-ACGUGACACGUUCGGAGAATT-3′) were transfected into the pancreatic cancer cells, respectively. Transfection of siRNA was performed using Lipofectamine™ 2000 (Invitrogen) according to the manufacturer's protocol. Briefly, the siRNA was blended with Opti-MEM® I Reduced Serum Media with the final concentration of 50 nmol/L. The mixture was transfected when the cells density reached about 30-50 percent. After culturing for 6 hours, the medium was changed with normal medium.

### Immunoblot analysis

The total protein was extracted using a RIPA lysis buffer (Beyotime). The cytosolic protein, mitochondrial protein and nuclear protein were extracted respectively using a mitochondria and nuclear protein extraction kit (Beyotime) according to the manufacturer's instructions. Equal amounts of proteins were loaded onto SDS-polyacrylamide gels and transferred onto a nitrocellulose membrane. The membrane was blocked with 5% non- fat milk powder in TBST for 1 h and incubated with primary antibodies against Bmi1 (1:500) (Santa Cruz), Bcl-2 (1:500) (CST), Bax (1: 1000) (CST), c-Myc (1:400) (Santa Cruz), Cyclin D1 (1:500) (CST), survivin (1:400) (Santa Cruz), Bcl-xL (1:400) (Santa Cruz), caspase 3 (1:1000) (CST), caspase 9 (1:300) (Bioss), COX IV (1:1000) (Abbkine). PARP-1 (1:1000), cytochrome-C (1:1000), Histone H3 (1:2000), phosphorylated IKKα/β (1:1000), IKKβ (1:1000), phosphorylated IκBα (1:1000), IκBα (1:1000), NF-κB/p65 (1:1000), GAPDH (1:1000), β-actin (1:1000) were purchased from CST. After washing with TBST, the membrane was incubated with secondary, horseradish peroxidase-coupled antibodies (Pierce) and visualized using enhanced chemiluminescence (Pierce). GAPDH or β-actin was used as internal controls.

### Quantitative real-time PCR assay

The treated cells were washed with PBS and the Total RNA was extracted using TRIzol (Invitrogen) according to the manufacturer's protocol. cDNA was obtained by reverse transcription with 1 μg of RNA with PrimeScript™ RT Master Mix (TaKaRa). QRT-PCR was performed according to the protocol of the quantitative SYBR Green PCR Kit (TaKaRa). Each reaction is set up in triplicate wells. GAPDH was used as internal controls. The relative expression of target gene was expressed with 2-^ΔΔCT^. Primer sequence (5′ to 3′): Bmi1-F: ACAAGACCAGACCACTACT, Bmi1-R: TCATTCACCTCCTCCTTAGA; CAT-F: ACATTACCAA ATACTCCAAGGCAAAG, CAT-R: AACCCGATTCTCC AGCAACA; MnSOD-F: ACCGAGGAGAAGTACCAG GAGG, MnSOD-R: ATTGATATGACCACCACCATT GAAC; GSTO1-F: CTACGAGTCTGCCATCAC, GS TO1-R: TGAGGTTCTGCCCGTTTGC; NQO1-F: ATATT GATGACTTCATGCCTGATTCC, NQO1-R: CAGAAC AGACTCGGCAGGATACT; SOD-F: GGAAGCATTAA AGGACTGACTGAAG, SOD-R: TTCTGGATAGAGGA TTAAAGTGAGGAC; GAPDH-F: GACGCTGGGGCTG GCATTG, GAPDH-R: GCTGGTGGTCCAGGGGTC.

### MTT Assay

After transfected with siRNA or plasmid for 36 h, followed by further gemcitabine treatment for 12 h, cells were planted in 96 well plates with a density of 7,000 cells/well and treated with increasing amounts of gemcitabine (Eli Lilly and Co.) for 48 h or 72 h. Then, 20μL of 3-(4, 5-Dimethyl-2-thiazolyl)-2, 5-diphenyl-2H-tetrazolium bromide (MTT) (Sigma) (5 mg/ml) was added and incubated for 4 h. The supernatant was replaced with 150 μl of dimethyl sulfoxide (Sigma) and read at 490nm using a microplate photometer. Every concentration had 5 replicate wells and each group was repeated thrice.

### ROS detection

The intracellular ROS was detected using a Reactive Oxygen Species Assay Kit (Beyotime) through a DCFH-DA probe. Briefly, the pancreatic cancer cells were harvested and washed with PBS, followed by incubating with DCFH-DA at a final concentration of 10 μM in serum-free medium for 20 min at 37°C. After the cells were washed three times and the fluorescence was measured using a fluorescence spectrophotometer.

### Immunofluorescence

Pancreatic cancer cells were plated in 6-well plates at a density of 2×10^4^ cells/well. After different treatment, the samples were fixed in 4% paraformaldehyde in PBS for 15 min at room temperature, then the samples were washed twice with ice cold PBS and permeabilized with 0.25% Triton-X. After blocked with 1% BSA in PBST for 30 min, the samples were incubated with the primary antibodies Bmi1 (1:50) (CST) in a humidified chamber for 1 hour at room temperature. After washed with PBST, the samples were incubated with the fluorescent secondary antibodies and DAPI. The slides were observed under fluorescence microscope.

### EMSA

Cells were washed twice with cold PBS, the nuclear protein was extracted and the protein concentration of nuclear extracts was determined using a Bio-Rad protein assay kit (Bio-Rad). Then, 10 μg of nuclear extract was used to detect the DNA-binding activity of NF-κB using a non-radioactive EMSA kit (Viagene Biotech). The sequence of the oligonucleotides used for the probe in the EMSA is 5′-AGTTGAGGGGACTTTCCCAGGC-3′.

### ELISA

The NF-κB DNA-binding activity was further determined by an ELISA based NF-κB p65 filter plate assay kits (Signosis), according to the manufacturer's instruction. The nuclear extract was added into the well pre-immobilized with the NF-κB consensus sequencing oligo. The activated NF-κB in nuclear extract is detected with a specific antibody against p65 subunit and an HRP conjugated secondary antibody subsequently. The optical density of each well was read at 450nm using a microplate photometer.

### Apoptotic detection

Pancreatic cancer cells were seeded in 6-well culture plates and subjected to different treatment. Before detection, all cells were collected including floating and attached cells and stained with Annexin V-FITC/PI according to the manufacturer's instructions (KeyGEN Biotech). Then the apoptotic cells were detected by flow cytometer analysis.

### Mitochondrial membrane potential (Δψm) measurement

The Δψm of pancreatic cancer cells was detected using a JC-1 Mitochondrial Membrane Potential Detection Kit (Beyotime) according to the manufacturer's protocol. Briefly, after harvested and trypsinzed into single cells (3×10^5^), pancreatic cancer cells were incubated with 2 μg/ml of JC-1 for 20 min at 37°C. And then after centrifugation and wash twice with the JC-1 dyeing buffer, the cells were suspended into the JC-1 dyeing buffer and detected by flow cytometry.

### Tumor xenograft

The significance of Bmi1 inhibition in the sensitization of gemcitabine in pancreatic cancer *in vivo* was studied by subcutaneous inoculation of cancer cells into the nude mice. PNAC-1 pancreatic cancer cells (5×10^6^ /100μl/mouse) were subcutaneously injected into the right flank of nude mice (n=5 for each variant). Tumor volume was calculated using the formula: length×width^2^/2. When the tumor volume reached approximately 120 mm^3^, the mice were randomly divided into 4 groups, namely Bmi1 siRNA, NC siRNA, GEM+Bmi1 siRNA and GEM+ NC siRNA, respectively.

For the gemcitabine treatment group, gemcitabine was intraperitoneally injected every 3 days with a dose of 10 mg/Kg. The Bmi1 siRNA or the NC siRNA was blended with the in vivo transfection reagent “Entranster™ -in vivo” (Engreen) with the ratio of 2:1. Transfection complexes were intratumoral injected at multiple points every 3 days for a total of 6 times. PBS was injected intraperitoneally as control. The tumor volume was monitored periodically (every 4 days). The laboratory animals were maintained under standard conditions and raised according to the National Research Council's guide for animal care.

The tumor xenografts were removed and fixed with 4% paraformaldehyde, paraffin embedded and sectioned at 5 μm. Bmi1 immunostaining was performed to detect the Bmi1 expression in pancreatic cancer tissues using Bmi1 antibody. Ki-67 immunostaining was performed to determine the proliferative activity of pancreatic cancer cells using an anti-Ki-67 monoclonal antibody (Saierbio). Apoptosis was measured by TUNEL assay using an apoptosis in situ detection kit (Beyotime).

### Statistical analysis

The results are expressed as mean ± SEM. Compa- risons between the two groups were evaluated using the Student's t test. All statistical analysis was performed using SPSS 18.0 software. P < 0.05 was considered significantly different.

## SUPPLEMENTARY FIGURES


