# Supplementary material for: Bmi1 inhibition enhances the sensitivity of pancreatic cancer cells to gemcitabine
Source: Oncotarget. 2016 May 11;7(24):37192–204. doi: 10.18632/oncotarget.9293 (PMC5095068; doi:10.18632/oncotarget.9293)
Supplement: Supplementary file 1 [file oncotarget-07-37192-s001.pdf]

Bmi1 inhibition enhances the sensitivity of pancreatic cancer cells to gemcitabine

SUPPLEMENTARY FIGURES

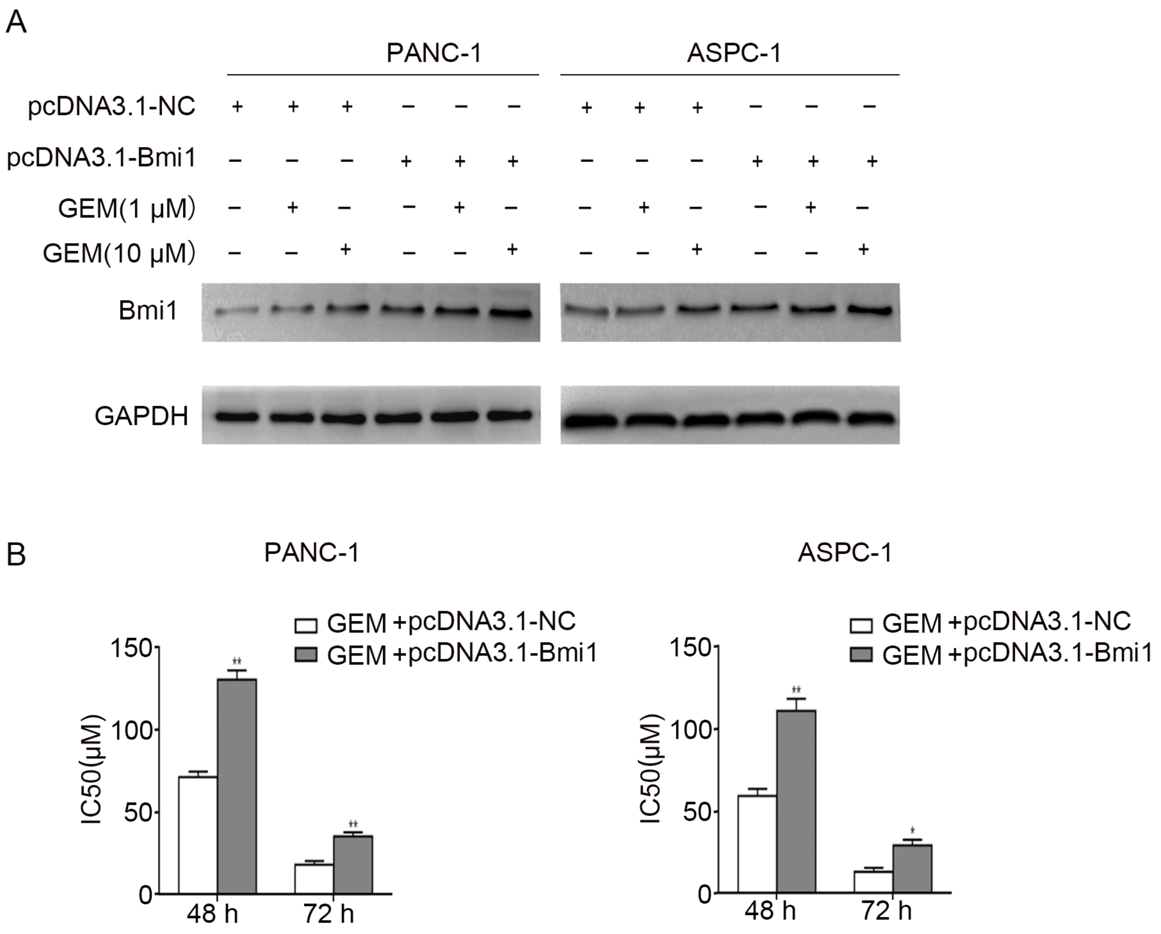

Supplementary Figure S1: Bmi1 overexpression promotes the expression of Bmi1 and increases the resistance of gemcitabine. A. PANC-1 and ASPC-1 cells were transfected with pCDNA3.1-Bmi1 and pCDNA3.1-NC for 36h, respectively, followed by different dose of gemcitabine treatments for 12h. The expressions of Bmi1 were detected by immunoblot analysis. Graphs shown are representative result of three independent assays. **B.** After treatment as indicated, the IC50s of gemcitabine at 48 h and 72 h in two cells was measured by MTT assays. The data were showed from 3 independent experiments *, $P < 0.05$; **, $P < 0.01$.

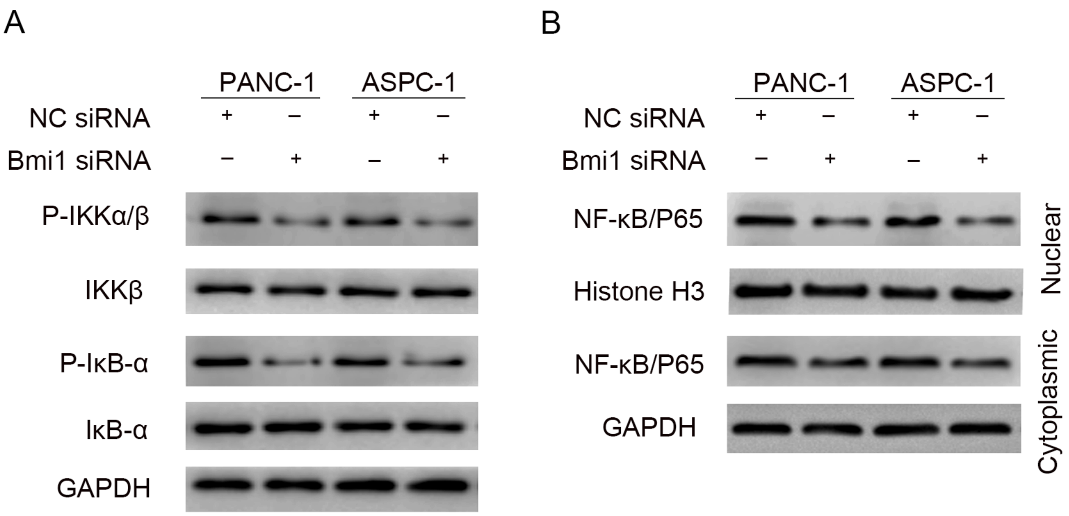

Supplementary Figure S2: Bmi1 knockdown inhibits the activation of NF-κB. A. After transfected with Bmi1 siRNA or NC siRNA for 48h, the expressions of phosphorylated IKKα/β, IKKβ, phosphorylated IκBα and IκBα were detected by immunoblot analysis. **B.** After above treatment, immunoblot analysis was used to examine the nuclear and cytoplasmic expression of NF-κB/p65.
